# Screening of Bacterial Strains for Polygalacturonase Activity: Its Production by *Bacillus sphaericus* (MTCC 7542)

**DOI:** 10.4061/2010/306785

**Published:** 2010-10-31

**Authors:** Ranveer Singh Jayani, Surendra Kumar Shukla, Reena Gupta

**Affiliations:** ^1^Department of Biotechnology, Himachal Pradesh University, Summer Hill, Shimla 171 005, India; ^2^Chromatin Biology Laboratory, National Centre for Cell Science (NCCS), Ganeshkhind, Pune 411 007, India

## Abstract

At present almost all the pectinolytic enzymes used for industrial applications are produced by fungi. There are a few reports of pectinase production by bacterial strains. Therefore, in the present study, seventy-four bacterial strains, isolated from soil and rotten vegetable samples, were screened for polygalacturonase production. The strain PG-31, which gave maximum activity, was identified as *Bacillus sphaericus* (MTCC 7542). Maximal quantities of polygalacturonase were produced when a 16-hours-old inoculum was used at 7.5% (v/v) in production medium and incubated in shaking conditions (160 rpm) for 72 hours. The optimal temperature and pH for bacterial growth and polygalacturonase production were found to be 30°C and 6.8, respectively. Maximum enzyme production resulted when citrus pectin was used as the carbon source at a concentration of 1.25% (w/v), whereas other carbon sources led to a decrease (30%–70%) in enzyme production. Casein hydrolysate and yeast extract used together as organic nitrogen source gave best results, and ammonium chloride was found to be the most suitable inorganic nitrogen source. The supplementation of media with 0.9% (w/v) D-galacturonic acid led to a 23% increase in activity. *Bacillus sphaericus*, a bacterium isolated from soil, produced good amount of polygalacturonase activity at neutral pH; hence, it would be potentially useful to increase the yield of banana, grape, or apple juice.

## 1. Introduction

Pectinolytic enzymes or pectinases are a heterogeneous group of enzymes that hydrolyze the pectic substances present in plants. They include polygalacturonases, pectin lyase, and pectin methyl esterase that hydrolyze the glycosidic bonds of pectic substances [1]. Endopolygalacturonase (EC 3.2.1.15) and exopolygalacturonase (EC 3.2.1.67) are the enzymes of particular interest to industry because they act on pectin, hydrolyzing its internal and external glycosidic bonds, producing shorter pectin molecular structures, decreasing the viscosity, increasing the yield of juices, and determining the crystalline structure of the final product [2]. 

At present almost all the pectinolytic enzymes used for industrial applications are produced by the fungi, namely,*. Aspergillus* sp., *Aspergillus japonicus*, *Rhizopus stolonifer*, *Alternaria mali*, *Fusarium oxysporum*, *Neurospora crassa*, *Penicillium italicum *ACIM F-152, and many others [3]. There are a few reports of pectinase production by bacterial strains. Some of the bacterial species producing pectinases are *Agrobacterium tumefaciens*, *Bacteroides thetaiotamicron*, *Ralstonia solanacearum,* and *Bacillus* sp. [3]. 

As we know the pectinases used in food industry are mainly produced from the different fungal species. Most of the fungal pectinases have optimum pH range between 3.0 and 6.0. This pH range is suitable for fruit juices, which have almost the same pH, but these enzymes are not suitable for the vegetable purees or the other preparations which need almost neutral pH range [4]. The objective of this work is to investigate the production of pectinolytic enzymes in bacterial strains isolated from soil and rotten vegetable samples, selecting the best species for polygalacturonase production and optimizing the culture conditions to maximize the enzyme production as well as their improved pH tolerance.

## 2. Materials and Methods

### 2.1. Chemicals and Reagents

Pectin (from citrus fruits), D-galacturonic acid monohydrate and casein acid hydrolysate (from bovine milk) were obtained from Sigma Chemicals Co. (St. Louis, MO, USA). Polygalacturonic acid was obtained from Lancaster Synthesis (Morecambe, England). All other chemicals were of analytical grade.

### 2.2. Microorganisms

Seventy-four bacterial strains were isolated in our laboratory by enrichment culture method, from the soil and rotten vegetable samples collected from Shimla, Himachal Pradesh, India. Pure cultures were repeatedly subcultured on agar plates and maintained for enzyme studies.

### 2.3. Production Media

The liquid medium (pH 6.8) used for bacterial growth and enzyme production was composed of 0.05% KCl, 0.1% MgSO_4_ · 7H_2_O, 0.1% *tri*-sodium citrate dihydrate, 0.1% citric acid, 0.1% yeast extract, 0.1% casein (acid hydrolysate), and 1.0% citrus pectin. Consecutive optimization of production media was carried out by altering, the cultivation conditions and the composition of the culture medium.

### 2.4. Screening of Isolates for Pectinolytic Activity

All the isolates were screened for polygalacturonase activity by culturing them in the liquid broth with the above-mentioned composition. For the production of pectinolytic enzymes, inoculum was prepared by inoculating 10 mL of sterilized media in test tubes with loop full of pure cultures and incubated at 160 rpm at 30°C. The inoculum (5% v/v) was transferred to 25 mL of production media in Erlenmeyer flasks (250 mL) and incubated for 72 hours at the culture conditions same as that for the inoculum. The broth was harvested using Whatman filter paper. Filtrate was used as crude enzyme for determining polygalacturonase activity.

### 2.5. Enzyme Assay

Polygalacturonase activity was determined by colorimetric method using polygalacturonic acid as substrate. One unit of enzyme activity was defined as the amount releasing one *μ*mol of galacturonic acid per minute under standard assay conditions. The reducing sugars released were measured by arsenomolybdate method of Nelson [5] and Somogyi [6].

### 2.6. Optimization Studies for Polygalacturonase Production

#### 2.6.1. Effect of Inoculum Age, Inoculum Size, and Incubation Time

Inoculum age was optimized by inoculating the production medium (secondary culture) with inocula (primary culture) of varying age, namely 8 hours, 12 hours, 16 hours, 20 hours, and 24 hours and assaying the filtered broth for enzyme activity. To study the effect of inoculum size, 2.5%, 5.0%, 7.5%, and 10% (v/v), inoculum was used to inoculate the production medium, and polygalacturonase activity was assayed in the broth. Effect of incubation time was studied by incubating the microorganism in production medium for different time intervals (24 hours, 48 hours, 72 hours, 96 hours, and 120 hours) and measuring the enzyme activity.

#### 2.6.2. Effect of Temperature and pH

Most favorable production temperature was studied by incubating the production medium at different temperatures (25°C, 30°C, 35°C, 40°C, and 45°C). Polygalacturonase activity was checked by using the standard assay method. For optimizing the production of pH, the production medium varying pH, namely, 4.4, 5.2, 6.0, 6.8, and 7.6, was used for enzyme production, and activity was measured.

#### 2.6.3. Effect of Carbon Sources

Various carbon sources citrus pectin, apple pectin, dextrose, fructose, galactose, lactose, maltose, sucrose, glycerol, and xylose were used in the production medium at a concentration of 1% w/v to check the effect of carbon source on enzyme production. The culture supernatants were assayed for polygalacturonase activity.

#### 2.6.4. Effect of Nitrogen Sources

The effect of various nitrogen sources (Ca(NO_3_)_2_, (NH_4_)_2_SO_4_, (NH_4_)NO_3_, NH_4_Cl, NaNO_3_, (NH_4_)_2_SO_4_FeSO_4_·6H_2_O, KNO_3_, (NH_4_)H_2_PO_4_, urea, casein hydrolysate, and yeast extract) on the production of enzyme was studied by supplementing 0.1% w/v of these to the production media.

#### 2.6.5. Effect of Concentration of Carbon Source

To study the effect of concentration of optimized carbon source for maximal enzyme production, it was used at different concentrations (0.5%, 0.75%, 1.0%, 1.25%, and 1.5% w/v) in the production media.

#### 2.6.6. Effect of D-Galacturonic Acid on Polygalacturonase Production

To study the effect of D-galacturonic acid on polygalacturonase production, it was added to the culture broth at a final concentration of 0.3, 0.6, 0.9, 1.2, and 1.5% w/v under aseptic conditions. The resulting extracellular polygalacturonase activities produced were measured by estimating the reducing groups produced.

#### 2.6.7. Growth and Enzyme Production Profile at Optimized Conditions

The growth and enzyme production profile of the microorganism was studied by withdrawing the samples from the culture flasks at regular intervals (8 hours) up to 120 hours. The supernatant was assayed for polygalacturonase activity. Also, absorbance of the culture was recorded at 600 nm to follow the growth pattern.

## 3. Results and Discussion

Out of the 74 bacterial isolates obtained, 9 showed the activity higher than 4.5 *μ*mol mL^−1^ min^−1^. Of these, PG-31, which showed the highest activity, was selected for further studies (Table 1). This strain was identified as *Bacillus sphaericus* (MTCC 7542). This strain showed more consistent results in repeated experiments, hence, PG-31 was selected for further studies. This is the first report on production of polygalacturonase from *Bacillus sphaericus.*

**Figure 1:**
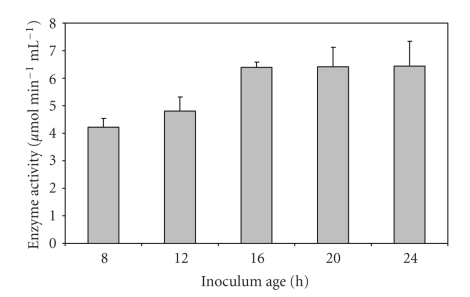
Effect of inoculum age on PGase production from *Bacillus sphaericus*.

**Figure 2:**
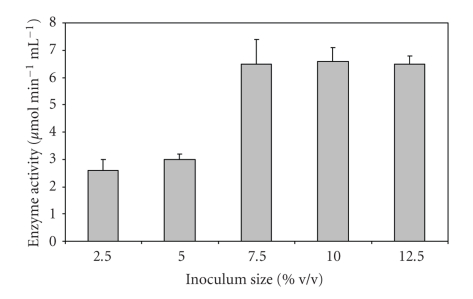
Effect of inoculum size on PGase production from *Bacillus sphaericus*.

**Figure 3:**
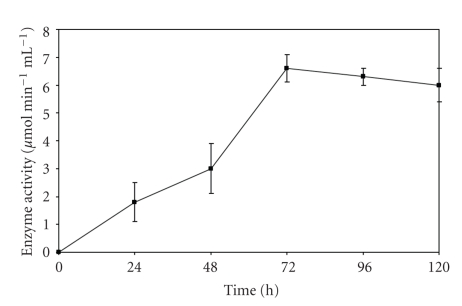
Effect of incubation time on PGase production from *Bacillus sphaericus*.

 A rapid increase in enzyme production was observed till the inoculum age of 16 h, and it became almost constant thereafter (Figure 1). The supernatant of the culture inoculated with 7.5% v/v inoculum gave the best polygalacturonase activity (Figure 2). As maximum enzyme production occurs in log phase of culture, an inoculum size of 7.5% v/v was able to provide enough biomass with an optimal length of log phase leading to higher levels of enzyme production. Further increase in inoculum size led to no increase in activity. Optimal incubation time for maximal PGase activity was found to be 72 hours (Figure 3). Loera et al. (1999) [7] also reported 73 hours to be optimum incubation time for maximal PGase activity by a diploid construct from two *Aspergillus niger* overproducing mutants. 

**Figure 4:**
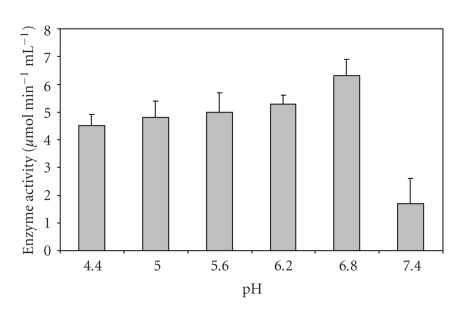
Effect of pH on PGase production from *Bacillus sphaericus*.

Most favorable production temperature for PGase production was found to be 30°C (Table 2). *Aspergillus* sp. ATHUM-3482, and *Peacilomyces clavisporus* 2A.UMIDA.1 also produced maximum PGase activity when incubated at 30°C for 72 hours [8]. The maximum enzyme activity was obtained when the initial pH of the production medium was adjusted to 6.8 (Figure 4), and there was a drastic decrease (80%) in enzyme activity at pH of 7.4. A pH range of 5.5–6.5 has been reported for maximum PGase production [9]. The pectinase produced by *Bacillus sphaericus* (MTCC 7542) shows almost neutral optimum pH, so this preparation can serve well for the vegetable purees or other preparations which need almost neutral pH range [4].

**Figure 5:**
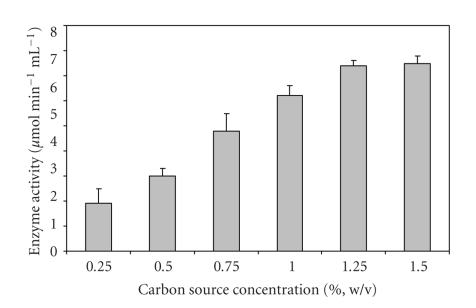
Effect of varying concentrations of carbon source (citrus pectin) on PGase production from *Bacillus sphaericus. *

**Figure 6:**
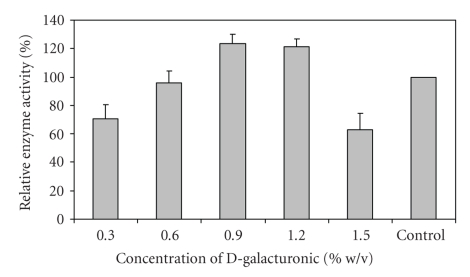
Effect of D-galacturonic acid on PGase production from *Bacillus sphaericus*.

Among the carbon sources used, citrus pectin gave the best activity for production of PGase by *Bacillus sphaericus* (Table 3). There was a loss of 34% activity when apple pectin was used, suggesting that the organism utilized citrus pectin more efficiently as compared to apple pectin. Of the various nitrogen sources used, maximum PGase activity was observed when casein hydrolysate and yeast extract were used together (Table 4). Among inorganic nitrogen sources, maximum activity (96% of control) was obtained with NH_4_Cl. Kashyap et al. (2003) [10] also reported that yeast extract and NH_4_Cl were found to enhance polygalacturonase activity by up to 24%. A loss of 80% activity was observed when (NH_4_)_2_SO_4_·6H_2_O was used as sole nitrogen source. An increase of 18% as compared to control (containing 1.0% w/v citrus pectin) was observed when pectin was used at a concentration of 1.25% w/v (Figure 5). It has been reported earlier that maximal PGase production in *Aspergillus niger* culture occurred on addition of 1.0% w/v pectin [11]. An increase of about 23% in activity was observed when the production medium was supplemented with 0.9% w/v D-galacturonic acid (Figure 6). Decreasing or increasing the concentration of D-galacturonic acid had an antagonistic effect on the production of the enzyme. PGase production is repressed on increasing or decreasing the concentration of D-galacturonic acid [12]. The stimulatory effect of the addition of D-galacturonic acid in the production medium on PGase production by *Sclerotinia sclerotiorum* [13] and *Aspergillus niger* [14] has been reported previously.

**Figure 7:**
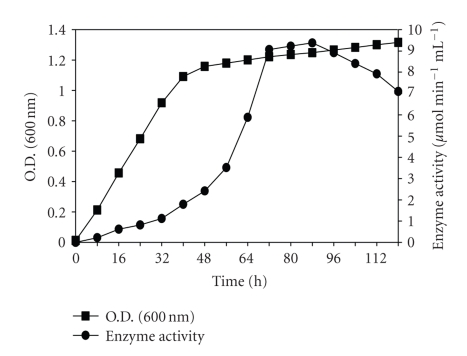
Growth and enzyme production profile of *Bacillus sphaericus. *

When the growth and enzyme production profile of *Bacillus sphaericus *was studied, a rapid increase in biomass during the first 48 hours of fermentation was observed, after which the growth became almost constant probably due to exhaustion of nutrients in the culture medium. The enzyme production started increasing after 48 hours and reached its maximum after a period of 72 hours. The results have been shown in Figure 7.

## 4. Conclusion

 In the present study, *Bacillus sphaericus, *a bacterium isolated from soil, produced good amount of polygalacturonase activity after 72 h of incubation in production medium at 30°C and pH 6.8. Maximum enzyme production was with citrus pectin as carbon source and with casein hydrolysate and yeast extract together as nitrogen source. This enzyme with good activity at neutral pH would be potentially useful to increase the yield of banana, grape, or apple juice.

## Figures and Tables

**Table 1:** Results of secondary screening: bacterial isolates showing higher activity.

S. no.	Strain no.	Enzyme activity (*μ*mol min^−1^ mL^−1^)
1	PG-21	4.9 ± 0.3
2	PG-31	6.2 ± 0.7
3	PG-34	4.3 ± 1.0
4	PG-47	5.2 ± 0.8
5	PG-Mp	4.8 ± 0.9
6	PG-P_XI_	4.5 ± 0.4
7	PG-A_3_	5.9 ± 1.1
8	PG-B_1-2_	4.4 ± 0.7
9	PG-B_2-2_	5.5 ± 0.8

Values are mean ± S.D. of 3 replicates.

**Table 2:** Effect of temperature on PGase production from *Bacillus sphaericus*.

Incubation temperature (°C)	Enzyme activity (*μ*mol min^−1^ mL^−1^)	Relative activity (%)
25	2.3 ± 1.0	37.1
30	6.2 ± 1.3	100.0
35	5.9 ± 1.7	95.8
40	5.4 ± 0.8	87.1
45	4.1 ± 0.9	65.9
50	1.7 ± 0.6	28.1

Values are mean ± S.D. of 3 replicates.

**Table 3:** Effect of various carbon sources on PGase production from *Bacillus sphaericus*.

Carbon source	Enzyme activity (*μ*mol min^−1^ mL^−1^)	Relative activity (%)
Citrus pectin	6.3 ± 0.7	100
Apple pectin	4.2 ± 1.1	66.8
Glucose	1.2 ± 0.3	20.1
Fructose	3.2 ± 0.8	50.9
Galactose	2.1 ± 0.5	32.9
Lactose	2.8 ± 0.2	45.3
Maltose	1.9 ± 0.8	29.8
Sucrose	3.9 ± 0.4	62.7
Glycerol	3.3 ± 0.7	52.3
Xylose	4.3 ± 0.6	67.9

Values are mean ± S.D. of 3 replicates.

**Table 4:** Effect of various nitrogen sources on PGase production from *Bacillus sphaericus*.

Nitrogen source	Enzyme activity (*μ*mol min^−1^ mL^−1^)	Relative activity (%)
(NH_4_)_2_SO_4_	4.0 ± 1.2	62.6
(NH_4_)NO_3_	5.1 ± 0.3	80.2
NH_4_Cl	6.2 ± 0.6	95.9
(NH_4_)_2_SO_4_·7H2O	1.2 ± 0.9	19.5
Ca(NO_3_)_2_	1.8 ± 0.3	28.3
KNO_3_	2.4 ± 0.7	37.6
NaNO_3_	2.6 ± 0.5	40.7
(NH_4_)H_2_PO_4_	3.4 ± 0.2	52.9
Urea	1.7 ± 0.6	26.9
Casein hydrolysate	5.0 ± 0.9	78.6
YE	6.0 ± 0.6	93.3
CH+YE (control)	6.4 ± 0.8	100.0

Values are mean ± S.D. of 3 replicates.
